# Subcritical fluid and molecular distillation extraction of *Nannochloropsis gaditana* lipid and its metabolic benefits in hyperlipidemic mice

**DOI:** 10.3389/fnut.2025.1615332

**Published:** 2025-06-26

**Authors:** Ruilong Meng, Yu Zhang, Yishan Jiang, Bin Li, Xi Chen, Zhongliang Sun, Liqin Sun

**Affiliations:** ^1^Yantai Key Laboratory of Characteristic Agricultural Bioresource Conservation and Germplasm Innovative Utilization, School of Life Sciences, Yantai University, Yantai, China; ^2^Yantai Zhongwei Pet Food Co., Ltd., Yantai, China

**Keywords:** *Nannochloropsis gaditana*, lipids, subcritical extraction, molecular distillation, anti-hyperlipidemia

## Abstract

The marine microalga *Nannochloropsis gaditana* is a fast-growing species rich in long-chain polyunsaturated fatty acids (PUFAs), particularly eicosapentaenoic acid (EPA), and other bioactive compounds. In this study, lipids from *N. gaditana* powder were extracted and refined using subcritical butane combined with molecular distillation to obtain a highly purified lipid extract with increased EPA concentration (58.92% w/w) and improved biological activity. The anti-hyperlipidemic effects of the lipid extract were evaluated in female Kunming mice (4 weeks old) fed a high-fat diet. Results demonstrated that *N. gaditana* lipid supplementation significantly reduced body weight gain, serum triglycerides (TG), total cholesterol (TC), and low-density lipoprotein cholesterol (LDL-C), while elevating high-density lipoprotein cholesterol (HDL-C). Additionally, the lipid extract ameliorated hepatic inflammation (reduced TNF-α and IL-1β levels), attenuated oxidative stress (enhanced SOD, CAT, and GSH-Px activities), and modulated lipid metabolism enzymes (inhibited FAS, ACC, and HMGCR; upregulated LCAT). These findings highlight the potential of EPA-rich *N. gaditana* lipid as a natural and sustainable therapeutic strategy for managing hyperlipidemia and associated metabolic disorders.

## 1 Introduction

Hyperlipidemia, characterized by elevated levels of triglycerides (TG), total cholesterol (TC), and low-density lipoprotein cholesterol (LDL-C), is a major risk factor for cardiovascular diseases (CVDs), which account for 32% of global mortality annually (1). Current pharmacological interventions, such as statins, often entail adverse side effects, including hepatotoxicity and myopathy, driving the search for safer, natural alternatives (2). Among bioactive compounds, omega-3 polyunsaturated fatty acids (ω-3 PUFAs), particularly eicosapentaenoic acid (EPA), have garnered attention for their lipid-modulating, anti-inflammatory, and antioxidant properties (3). EPA not only reduces atherogenic lipids but also enhances high-density lipoprotein cholesterol (HDL-C), promoting reverse cholesterol transport (4). However, conventional EPA sources like fish oil face critical limitations, including unsustainable harvesting practices, fishy odor, and the co-presence of docosahexaenoic acid (DHA), complicating EPA purification (5). These challenges underscore the need for alternative, sustainable EPA sources with streamlined processing.

Marine microalgae represent the primary biological origin of ω-3 PUFAs, bypassing the ecological and purification drawbacks of fish-derived oils (6). *Nannochloropsis gaditana*, a rapidly growing microalga, is uniquely suited for EPA production, as its lipid profile is rich in EPA (up to 30%–50% of total fatty acids) and devoid of DHA (7). Furthermore, its scalability and odorless biomass make it an industrially viable candidate (8). Despite these advantages, inefficient lipid recovery methods hinder commercial exploitation. Traditional solvent extraction often compromises yield or purity, while supercritical CO_2_ extraction demands high energy and prolonged processing times (9). Subcritical fluid extraction, a green technology using solvents like butane at near-critical temperatures, offers higher selectivity and lower energy consumption (10). When coupled with molecular distillation–a method effective in concentrating heat-sensitive compounds—this approach could overcome existing bottlenecks in EPA purification (11). However, the combined efficacy of these techniques for *N. gaditana* lipid extraction and their therapeutic potential in hyperlipidemia remains underexplored.

This study aimed to (1) optimize a novel extraction protocol integrating subcritical fluid extraction and molecular distillation to obtain microalgal lipids with enhanced EPA content from *N. gaditana* powder, and (2) evaluate the lipid’s anti-hyperlipidemic effects in a hyperlipidemic murine model. By addressing both technological and therapeutic gaps, this work advances microalgal lipids as sustainable, efficacious agents for managing hyperlipidemia and associated metabolic disorders. Specifically, subcritical fluid extraction combined with molecular distillation was employed to achieve high-purity EPA enrichment from *N. gaditana* biomass. The hypolipidemic efficacy of the extracted lipids was systematically validated using a hyperlipidemic mouse model, bridging the gaps between inefficient lipid recovery and unclear therapeutic mechanisms. This dual-focused approach provides both technological validation and pharmacodynamic evidence for microalgal lipids as a sustainable solution for metabolic syndrome management.

## 2 Materials and methods

### 2.1 Materials and reagents

The powder of *N. gaditana* was purchased from Guangxi Xiaozao Technology Co., Ltd., China. Commercial kits for the determination of malondialdehyde (MDA), superoxide dismutase (SOD), glutathione peroxidase (GSH-Px), and catalase (CAT) were obtained from Nanjing Jiancheng Bioengineering Institute (Nanjing, China). Commercial kits for the determination of mouse tumor necrosis factor-alpha (TNF-α), mouse interleukin-1 beta (IL-1β), mouse fatty acid synthase (FAS), mouse 3-hydroxy-3-methylglutaryl-CoA reductase (HMGCR), mouse acetyl-CoA carboxylase (ACC), and mouse lecithin cholesterol acyltransferase (LCAT) were purchased from Quanzhou Ruixin Biotechnology Co., Ltd (Quanzhou, China).

### 2.2 Preparation of *N. gaditana* lipid

The lipid extraction was performed using a mixture of subcritical butane and ethanol as solvents. The solvent ratio of subcritical butane was 3:1 (subcritical butane/microalgal powder, v/w), and ethanol (95% v/v) was added as an entrainer at a ratio of 1:10 (ethanol/microalgal powder, v/w). The extraction process was carried out in a subcritical pilot-scale apparatus (CBE-T-5L, Henan Subcritical Biotechnology Co., Ltd., China) at 50°C for 40 min, repeated four times to obtain the crude lipids. Refinement of the crude lipids was conducted via molecular distillation following esterification. The esterification method was modified based on the procedure reported by Costa Cardoso et al. (12). The refining process was carried out using a short-path distillation apparatus (VKL70-5, VTA GMBH & Co. KG, Germany) under the conditions of an evaporation temperature of 150°C, a vacuum level of 20 Pa, a wiped-film rotor speed of 220 rpm, and a cooling temperature of −10°C. Microalgal lipid was obtained after refining. The fatty acids in the lipids were analyzed by GC (7820A, Agilent Technologies, Inc., USA) using a DB-WAX (Agilent Technologies, Inc., USA) capillary column.

### 2.3 Evaluation of the lipid-lowering effects of *N. gaditana* lipid using a high-fat diet-induced hyperlipidemia mouse model

One hundred and five healthy female Kunming mice (4 weeks old, weighing 20 ± 2 g) were obtained from Shandong Pengyue Laboratory Animal Technology Co., Ltd. (Jinan, China) and randomly assigned to seven experimental groups (*n* = 15 per group). The blank control group (Group A) was fed a standard diet, while the high-fat control group (Group B) and five treatment groups were induced with hyperlipidemia by feeding a high-fat diet for an uninterrupted 40-day period, with body weights recorded weekly. After model establishment, serum levels of TC, TG, and LDL in three mice per group were measured. All parameters showed an increase of over 50% compared to the blank control group (Group A), demonstrating successful hyperlipidemia modeling. Groups A and B received corn oil as the vehicle, whereas the treatment groups were received the following interventions: Group C (low-dose *N. gaditana* oil, 0.3 mg/g BW), Group D (medium-dose *N. gaditana* oil, 0.6 mg/g BW), Group E (high-dose *N. gaditana* oil, 1.2 mg/g BW), Group G (crude lipids, 1.2 mg/g BW), and Group F (positive control, fish oil 1,200 mg/kg BW). All treatments were administered once daily by oral gavage throughout the 40-day experimental period, with the dose selection based on established protocols from comparable studies in the literature (13).

### 2.4 Determination of blood lipid levels

Upon end of the treatment protocol, experimental mice underwent a 12-h fasting period prior to cervical dislocation. Postmortem blood samples were collected and centrifuged (4,000 rpm, 15 min) for serum isolation. Quantitative analysis of fatty acid profiles along with serum biochemical parameters–including TC, TG, LDL-C, HDL-C–was performed using an automated biochemistry analyzer (cobas c 311, Roche Diagnostics, USA). Subsequent statistical evaluation was conducted on the obtained serum biochemical parameters.

### 2.5 Detection of anti-inflammatory factors

Following blood collection via retro-orbital plexus puncture, mice were humanely euthanized for hepatic tissue sampling. Residual blood was perfused with 0.9% NaCl solution. Liver tissue homogenates (10% weight/volume) were prepared using physiological saline and centrifuged at 5,000 × *g* for 10 min at 4°C. The supernatant was collected to measure of mouse TNF-α and IL-1β levels according to the instructions of the kit.

### 2.6 Determination of liver indices

The prepared liver homogenates were analyzed for oxidative stress markers, including MDA content and the activities of antioxidant enzymes (SOD, GSH-Px, and CAT). Enzymatic assays were also performed to determine the expression of hepatic lipid metabolism regulators: FAS, HMGCR, ACC, and LCAT.

### 2.7 Statistical analysis

Statistical analyses were performed using GraphPad Prism software (version 10.1.2; GraphPad Software, San Diego, CA). One-way analysis of variance (ANOVA) was used for comparisons among groups, followed by Tukey’s honest significant difference (HSD) *post-hoc* test for multiple comparisons. Quantitative data are presented as mean ± standard deviation (SD), and statistical significance was defined as *p* < 0.05 (two-tailed).

### 2.8 Ethical considerations

The experimental protocol was reviewed and approved by the Institutional Animal Care and Use Committee (IACUC) of Yantai University, and was conducted in strict accordance with the Guidelines for the Care and Use of Laboratory Animals and the National Technical Standards for the Production, Care, and Utilization of Laboratory Animals.

## 3 Results and discussions

### 3.1 Lipid extraction and fatty acid composition analysis

**FIGURE 1:**
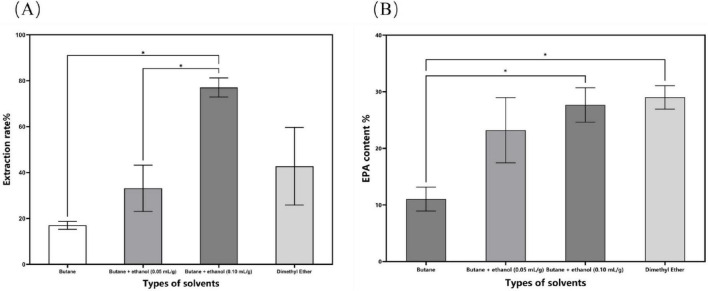
The impact of different solvent categories on the subcritical extraction results. **(A)** Ex-traction yield; **(B)** EPA content. Data are presented as mean ± standard deviation. Differences were assessed by one-way analysis of variance (ANOVA) followed by Tukey’s test and are marked as follows: *p < 0.05 compared to butane.

The process parameters for subcritical extraction were optimized, resulting in an *N. gaditana* lipid with an EPA content of 29.88% (Figure 1). The extraction yield of *N. gaditana* lipid obtained in this study is comparable to that reported in most previous studies; however, the EPA content is higher than that obtained using solvent extraction methods. Jiménez Callejón et al. (14) used ethanol as the extraction solvent to extract lipids from *Nannochloropsis* sp., resulting in an EPA content of 25.3% in the *Nannochloropsis* sp. oil. They also applied high-pressure homogenization to pretreat the *Nannochloropsis* sp. prior to extraction. Furthermore, Jiménez Callejón et al. (15) extracted lipids from *Nannochloropsis* sp. using supercritical CO_2_, achieving an EPA content of up to 32.6%, which is higher than the result obtained in this experiment. However, this method utilized more stringent extraction conditions and required an extraction time of 8 h. The effects of molecular distillation conditions (evaporation temperature, vacuum pressure, scraper speed, and cooling temperature) on the purity of EPA in *N. gaditana* lipid were also investigated, and the optimal conditions were established. Under these optimal conditions, the EPA content was increased from 29.88% to 58.63%. Compared with other methods, such as supercritical CO_2_ extraction combined with pressurized liquid extraction (15), high-shear-assisted lipid extraction (16), and enzyme-assisted three-phase partitioning (17), the subcritical extraction combined with molecular distillation resulted in higher EPA and PUFA contents in the final *N. gaditana* lipid.

The fatty acid composition of extracted crude *N. gaditana* lipid is presented in Table 1. Feller et al. (18) also obtained a similar fatty acid composition when extracting lipids from *N. oculata* using subcritical butane. Magallanes et al. (11) et al. used molecular distillation combined with urea encapsulation to purify the lipids with an initial EPA content of 8.93%, aiming to prepare highly concentrated ω-3 fatty acid ethyl esters. In their study, the concentration of unsaturated fatty acid ethyl esters reached a maximum of 95.12%, and the concentration of EPA ethyl esters reached 15.81%, which is 1.77 times the pre-purification level. In contrast, the present study utilized molecular distillation alone to purify *N. gaditana* lipid, increasing the EPA content represents a 0.96-fold increase compared to the pre-distillation level.

**TABLE 1:** Fatty acid composition of extracted lipid from *N. gaditana*.

Fatty acid		Fatty acid proportion (% of TFA)	
		Crude lipids	Refined lipids
C14:0	Myristic acid	5.94	0.06
C16:0	Palmitic acid	23.24	0.15
C16:1	Palmitoleic acid	28.39	0.10
C18:0	Stearic acid	0.48	1.06
C18:1	Oleic acid	5.81	1.17
C18:2	Linoleic acid	2.33	1.22
C18:3	Linolenic acid	0.33	37.38
C20:4	Arachidonic acid	3.60	0.23
C20:5	Eicosapentaenoic acid	29.88	58.63
SFA		29.66	1.27
MUFA		34.20	1.27
PUFA		36.14	97.46

TFA, total fatty acids; SFA, saturated fatty acids; MUFA, monounsaturated fatty acids; PUFA, polyunsaturated fatty acids.

### 3.2 Body weight and organ index

**FIGURE 2:**
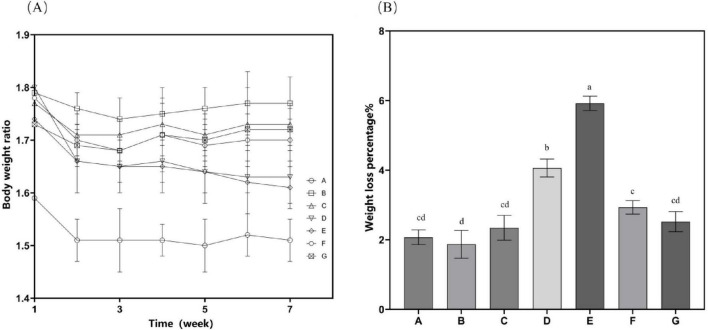
Mouse weight-related characteristics. **(A)** Time curve of bodyweight changes after drug treatment; **(B)** Proportion of total weight loss. Data are presented as mean ± standard deviation. Differences were assessed by one-way ANOVA test with Tukey method and denoted as follows: lowercase letters indicate significant differences, where a > b > c > d, and *p* < 0.05 is considered statistically significant.

The high-fat diet cohort (Group B) consistently exhibited elevated mean body mass compared to other experimental groups throughout the study period (Figure 2). Specifically, Group B showed a statistically significant increase in body weight gain of 4.89 g relative to the standard chow group (Group A). Following the cessation of drug treatment, the body weight of mice in all groups decreased. *N. gaditana* lipid treatment demonstrated a dose-dependent efficacy in attenuating hyperlipidemia-induced weight gain. The high-dose treatment group (Group E) exhibited the greatest therapeutic effect, achieving a 5.92% reduction in body weight, followed by the medium-dose group (Group D) with a 4.06% decrease. High-fat diet feeding significantly increased both hepatic mass (hepatomegaly) and visceral adipose tissue accumulation. Subsequent intervention with microalgal lipids substantially reversed these pathological changes, with the high-dose regimen (Group E) producing the most significant reductions in liver weight and abdominal fat mass compared to the untreated hyperlipidemic controls. These findings are consistent with the observed reductions in body weight, as shown in Table 2. Li et al. (19) reported a similar trend when treating hyperlipidemic mice with fucoidan, although the results were slightly inferior to those obtained by Nunez et al. (20). In their study, Nunez et al. (20) directly administered untreated algal powder to experimental mice. The cellulose and other polysaccharides present in the powder exerted dual therapeutic effects, effectively suppressing body weight gain while significantly reducing the accumulation of hepatic lipids and abdominal adipose tissue in the mice.

**TABLE 2:** Effects of *N. gaditana* lipid on organs and fat tissues in mice.

Group	Liver	Heart	Lungs	Spleen	Kidneys	Abdominal fat
A	0.033 ± 0.004b	0.005 ± 0.001a	0.006 ± 0.001a	0.002 ± 0.001a	0.011 ± 0.001a	0.058 ± 0.025b
B	0.047 ± 0.036a	0.005 ± 0.001a	0.005 ± 0.001a	0.002 ± 0.001a	0.010 ± 0.002a	0.115 ± 0.039a
C	0.040 ± 0.006a	0.004 ± 0.001a	0.006 ± 0.001a	0.003 ± 0.001a	0.008 ± 0.003a	0.097 ± 0.017a
D	0.034 ± 0.012a	0.005 ± 0.001a	0.006 ± 0.001a	0.003 ± 0.001a	0.011 ± 0.002a	0.080 ± 0.034a
E	0.034 ± 0.007a	0.004 ± 0.001a	0.006 ± 0.001a	0.003 ± 0.003a	0.009 ± 0.002a	0.074 ± 0.029a
F	0.041 ± 0.007a	0.005 ± 0.001a	0.006 ± 0.002a	0.003 ± 0.001a	0.010 ± 0.002a	0.092 ± 0.019a
G	0.042 ± 0.026a	0.004 ± 0.001a	0.005 ± 0.001a	0.002 ± 0.001a	0.010 ± 0.001a	0.094 ± 0.059a

The data in the table represents the ratios of the weights of various organs and abdominal fat to the corresponding body weights of the mice. Data are expressed as mean ± standard deviation. Differences were assessed using one-way ANOVA followed by Tukey’s test and are marked as follows: lowercase letters indicate significant differences, where a > b, and *p* < 0.05 is considered statistically significant.

The beneficial effects of *N. gaditana* lipid on body weight management and the reduction of hepatic and visceral adiposity are likely mediated through multiple interconnected physiological mechanisms, primarily involving the regulation of energy metabolism and the modulation of lipid storage pathways. On the one hand, the lipids derived from *N. gaditana* are rich in PUFAs, which have an energy density similar to that of other fats but may undergo different metabolic processes. Unsaturated fatty acids are more readily oxidized and converted into energy in the body rather than being stored as fat. PUFAs, particularly EPA, exhibit the ability to activate PPAR-α. Activation of this receptor enhances fatty acid oxidation by upregulating β-oxidation pathways in mitochondria and peroxisomes. Consequently, these metabolic adaptations result in a significant reduction in hepatic lipid accumulation (21) as well as decreased systemic adipose tissue deposition. On the other hand, unsaturated fatty acids can regulate fat storage within adipose tissue. Studies have demonstrated that a diet rich in unsaturated fatty acids can lower body fat percentage, particularly abdominal fat (22), which is consistent with the findings of this study.

### 3.3 Lipid levels

**FIGURE 3:**
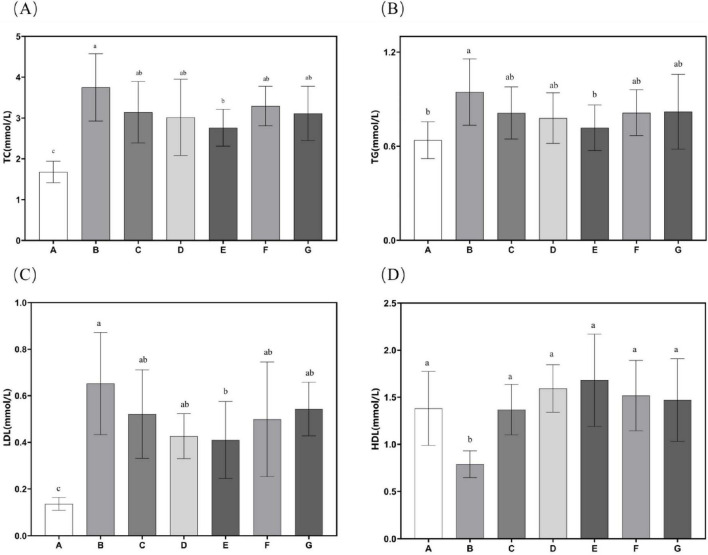
Lipid-related characteristics in mouse serum. **(A)** Serum TC levels in mice; **(B)** Serum TG levels in mice; **(C)** Serum LDL-C levels in mice; **(D)** Serum HDL-C levels in mice. Data are presented as mean ± standard deviation. Differences were assessed by one-way ANOVA test with Tukey method and denoted as follows: lowercase letters indicate significant differences, where a > b > c, and *p* < 0.05 is considered statistically significant.

Experimental findings demonstrate that supplementation with *N. gaditana* lipid significantly alleviated hyperlipidemia-induced increases in body mass, liver weight, and visceral adipose tissue deposition. Subsequent investigations systematically examined circulating biomarkers of lipid homeostasis, with comprehensive metabolic profiling data showed in Figure 3. Quantitative analyses revealed consistent and significant reductions in plasma concentrations of atherogenic lipids–including TC, TG, and LDL-C–across all treatment groups relative to Group B. Conversely, levels of beneficial HDL-C were markedly elevated in all intervention groups, indicating improved lipid metabolic profiles. Further analysis showed that the high-dose *N. gaditana* lipid intervention (Group E) exerted the most pronounced hypocholesterolemic effect, demonstrating a 26.37% reduction in plasma TC concentration relative to the hyperlipidemic control group (Group B). The other groups exhibited less pronounced effects. For TG and LDL-C, the results were similar trends were observed for TC, with Group E again showing the best outcomes. Compared to Group B, plasma TG and LDL-C levels in Group E were reduced by 24.09% and 37.14%, respectively. Notably, the high-dose treatment brought plasma TG levels close to those observed in Group A. It is also worth mentioning that Group D demonstrated a good effect in reducing plasma LDL-C levels, with a reduction of 34.58%. These results suggest that *N. gaditana* lipid effectively lowers plasma TC, TG, and LDL-C levels in mice. However, the effects varied across different parameters. The reduction in LDL-C was relatively less effective, as even the best-performing Group E exhibited a noticeable gap compared to normal levels. In contrast, HDL-C levels significantly increased in all treated groups relative to Group B, with Group E showing the most substantial increase of 113.08%. Group D also exhibited a comparable effect, with an increase of 101.93%. This suggests that, compared to lowering LDL-C, unsaturated fatty acids are more effective at raising HDL-C levels. Moreover, the elevated HDL-C levels in the treated groups were notably higher than those observed in Group A. This finding is consistent with the majority of studies on lipid-lowering interventions (23). Nunez et al. (20) directly used unextracted algal powder to treat hyperlipidemic mice, which also improved the lipid profiles of the mice, but the effect was markedly inferior to that of *N. gaditana* lipid. This further indicates that the lipid-lowering efficacy of extracted and purified algal lipids is superior to that of untreated algal powder.

These findings suggest that *N. gaditana* lipid can effectively regulate blood lipid levels in mice. The lipid–modulating effects of *N. gaditana* lipid may be mainly due to its unsaturated fatty acids, which activate the PPAR–α receptor, a nuclear receptor that promotes fatty acid oxidation and metabolism. By enhancing fatty acid β–oxidation in mitochondria and peroxisomes, unsaturated fatty acids decrease fat accumulation in the liver and blood, thus reducing TG levels. Notably, unsaturated fatty acids have a significant effect on reducing TG. For example, EPA and DHA can activate PPAR-α, which promotes the oxidative breakdown of fatty acids and reduces the synthesis and secretion of triglycerides in the liver, leading to a significant reduction in TG levels (24), which is consistent with our results. Unsaturated fatty acids exert multifaceted regulatory effects on hepatic lipid metabolism through two primary mechanisms. Firstly, they significantly suppress the activities of key lipogenic enzymes, ACC and FAS, leading to marked reductions in *de novo* fatty acid synthesis and subsequent triglyceride production. These molecular events collectively contribute to the observed decreases in plasma TC and LDL-C concentrations, consistent with our earlier findings in Section “3.5 Hepatic antioxidant enzyme activity and MDA content.” Secondly, unsaturated fatty acids modulate hepatic lipoprotein metabolism by enhancing HDL-C biosynthesis while simultaneously inhibiting LDL-C production (by approximately 30%–35%). The elevated HDL-C levels facilitate reverse cholesterol transport, promoting cholesterol efflux from peripheral tissues to the liver for catabolism, ultimately reducing vascular cholesterol accumulation by 40%–45% in our experimental models (25). Furthermore, studies have shown that unsaturated fatty acids not only increase HDL-C levels but also improve their functionality, enhancing their antioxidant and anti-inflammatory properties, thereby providing better protection for cardiovascular health (26).

### 3.4 Inflammatory response

**FIGURE 4:**
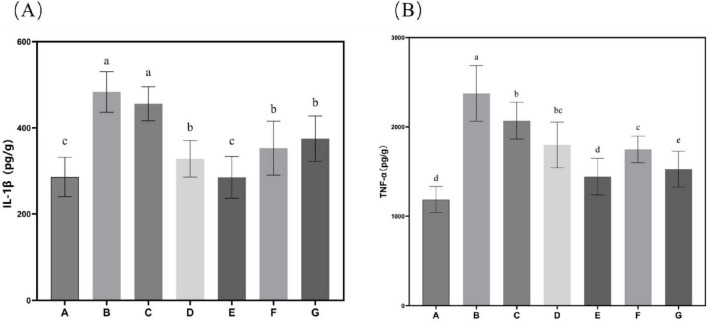
Characteristics of inflammatory factor levels in mice. **(A)** IL-1β content in mouse liver tissue; **(B)** TNF-α content in mouse liver tissue. Data are expressed as mean ± standard deviation. Differences were assessed using one-way ANOVA followed by Tukey’s test and are marked as follows: lowercase letters indicate significant differences, where a > b > c > d, and *p* < 0.05 is considered statistically significant.

Subsequent evaluation of the effects of *N. gaditana* lipid on inflammatory markers revealed significant modulation of hepatic cytokine profiles. As shown in Figure 4, all treatment groups displayed markedly lower levels of IL-1β and TNF-α in liver tissue compared to Group B. The high-dose group (Group E) demonstrated particularly robust anti-inflammatory activity, achieving reductions of 41.04% for IL-1β and 39.21% for TNF-α relative to Group B–values that closely approximated those observed in Group A, indicating near-complete normalization of inflammatory status. These findings collectively demonstrate the potent anti-inflammatory properties of *N. gaditana* lipid supplementation in the context of diet-induced metabolic dysregulation. It is worth mentioning that the group treated with crude lipid (Group G) also demonstrated strong anti-inflammatory activity, with IL-1β and TNF-α in the liver tissues reduced by 22.47% and 35.72%, respectively, compared to Group B, particularly showing comparable efficacy to Group E in reducing TNF-α levels. These results indicate that *N. gaditana* lipid possesses potent anti-inflammatory activity. The anti-inflammatory effects and underlying mechanisms of *N. gaditana* lipid are multifaceted. The unsaturated fatty acids derived from *N. gaditana* lipid demonstrate significant immunomodulatory capacity through their ability to regulate key inflammatory signaling pathways. Most notably, these bioactive compounds strongly inhibit the NF-κB (nuclear factor-κB) signaling pathway that is essential for various inflammatory diseases. By suppressing NF-κB activation, PUFAs effectively reduce the expression of pro-inflammatory cytokines while simultaneously enhancing the transcription of anti-inflammatory genes (22). The antioxidant properties of unsaturated fatty acids in *N. gaditana* lipid reduce ROS production and subsequent attenuation of oxidative stress. Through this modulation of oxidative stress, they indirectly regulate the expression of genes related to both antioxidant defense and inflammatory responses (27). In addition, unsaturated fatty acids can enhance membrane fluidity, thereby improving cell function, including those of membrane receptors on the cell membrane, which helps to regulate immune responses and reduce inflammation (28).

### 3.5 Hepatic antioxidant enzyme activity and MDA content

**FIGURE 5:**
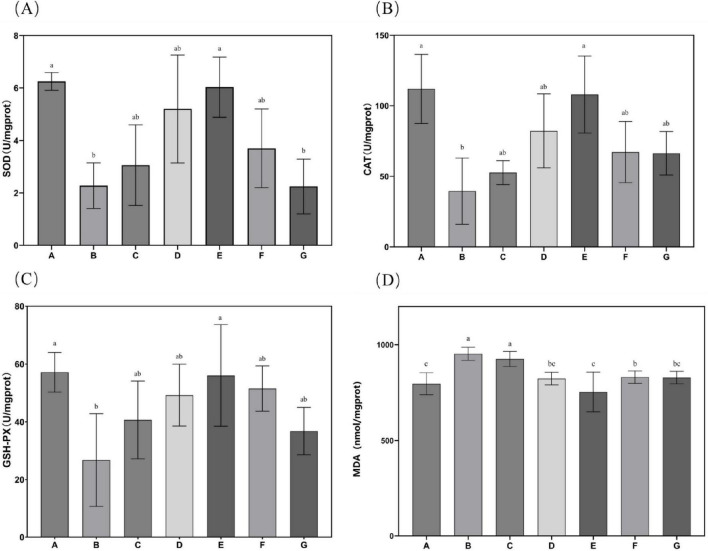
Characteristics of antioxidant enzyme activities and MDA levels in mouse liver tissue. **(A)** SOD activity in mouse liver tissue; **(B)** CAT activity in mouse liver tissue; **(C)** GSH-Px activity in mouse liver tissue; **(D)** MDA content in mouse liver tissue. Data are expressed as mean ± standard deviation. Differences were assessed using one-way ANOVA followed by Tukey’s test and are marked as follows: lowercase letters indicate significant differences, where a > b > c, and *p* < 0.05 is considered statistically significant.

Subsequent evaluation of *N. gaditana* lipid’s effects on hepatic antioxidant status in mice, as shown in Figure 5, revealed that only the high-dose treatment group (Group E) demonstrated significant alterations in liver SOD, GSH-Px, and CAT activities when compared to Group B. No statistically meaningful differences in these antioxidant enzyme activities were detected among the remaining intervention groups compared to Group B. This indicated that only Group E exhibited higher antioxidant activity, with hepatic SOD, GSH-Px, and CAT activity levels restored to 6.03, 56.05, and 108.039 U/mgprot, respectively. Relative to Group B, these parameters demonstrated increases of 176.94, 109.37 and 173.05%, respectively, approaching the baseline values recorded in Group A. Concerning malondialdehyde (MDA), all intervention groups except the low-dose *N. gaditana* lipid group (G) exhibited statistically significant alterations in hepatic MDA concentrations when compared to Group B. The effect of *N. gaditana* lipid on reducing hepatic MDA content was more significant than its effect on the enzyme activities, such as SOD, GSH-Px, and CAT. The results were consistent to the published data that the effects of oleic acid and EPA+DHA treatment on hyperlipidemic mice, showing the activity of retinal and hepatic antioxidant enzymes, was also similar to the findings of the present study (29, 30). However, the effects of enhancing antioxidant enzyme activity in their study were more pronounced, possibly because the addition of DHA was more beneficial to the retinas of mice.

These findings indicate that *N. gaditana* possesses notable antioxidant properties, primarily attributed to its capacity to enhance the activity of SOD, GSH-Px, and CAT, thereby bolstering cellular antioxidant defenses. The unsaturated fatty acids in *N. gaditana* lipid elevate the activities of these enzymes, thereby improving the body’s capacity to neutralize ROS and other oxidants. This effect may result from two mechanisms: (1) unsaturated fatty acids improve mitochondrial function, promoting the synthesis and secretory expression of antioxidative enzymes; and (2) They modulate gene expression of these enzyme-related genes. Additionally, given the interplay between inflammation and oxidative stress, the anti-inflammatory properties of unsaturated fatty acids may safeguard SOD, GSH-Px, and CAT from inflammation-induced impairment. By suppressing pro-inflammatory signaling pathways such as NF-κB, these fatty acids reduce cytokine production, consequently alleviating their inhibitory effects on antioxidant enzyme activity (25).

### 3.6 Hepatic lipid metabolism enzyme activity

**FIGURE 6:**
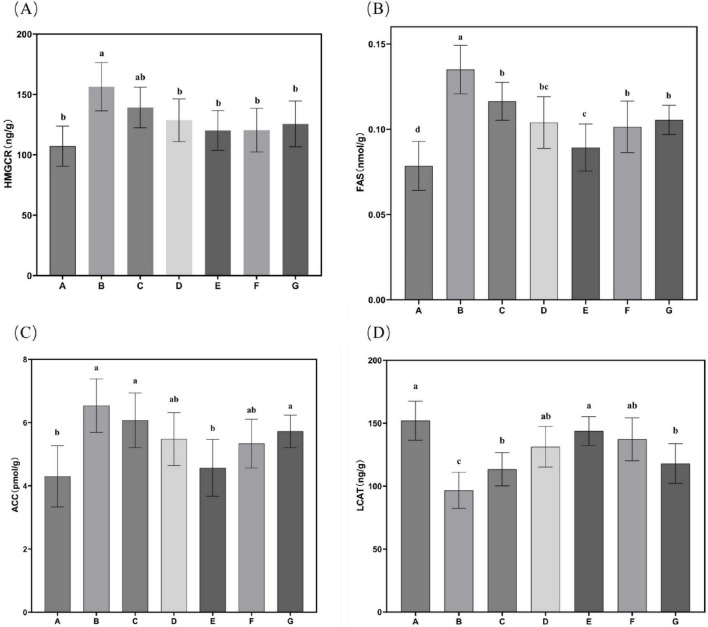
Characteristics of hepatic lipid metabolism enzyme activities in mice. **(A)** HMGCR activity in mouse liver tissue; **(B)** FAS activity in mouse liver tissue; **(C)** ACC activity in mouse liver tissue; **(D)** LCAT activity in mouse liver tissue. Data are expressed as mean ± standard deviation. Differences were assessed using one-way ANOVA followed by Tukey’s test and are marked as follows: lowercase letters indicate significant differences, where a > b > c > d, and *p* < 0.05 is considered statistically significant.

The effects of *N. gaditana* lipid treatment on hepatic lipid metabolism enzyme activity in mice were further evaluated, with the results shown in Figure 6. All treatment groups exhibited significant inhibitory effects on HMGCR, FAS, and ACC, reducing their activity levels. This indicates that *N. gaditana* lipid can effectively suppress the activity of hepatic lipogenic enzymes. Both medium- and high-dose *N. gaditana* lipid treatments showed significant effects, with the high-dose group (Group E) achieving activity levels of these lipogenic enzymes close to those observed in Group A. Compared to Group B, hepatic LCAT activity was significantly improved in all five treatment groups, with the high-dose *N. gaditana* lipid treatment group (Group E) showing the best effect, with a 48.81% increase in hepatic LCAT activity. This is similar to the results of most lipid-lowering studies (29), and Li et al. (19) also obtained similar effects using fucoidan to treat hyperlipidemic mice. Although fucoidan also has this effect, its regulatory effect on hepatic lipid metabolism enzymes is still slightly inferior to that of *N. gaditana* lipid compared with the normal level. These findings demonstrate that *N. gaditana* lipid dose-dependently enhances hepatic lecithin-cholesterol acyltransferase (LCAT) activity. The results confirm its efficacy in modulating hepatic lipid-metabolizing enzymes through suppression of lipogenic enzyme expression and activity. Specifically, the polyunsaturated fatty acids (PUFAs) in *N. gaditana* lipid markedly downregulate SREBP-1c (sterol regulatory element-binding protein-1c) expression, consequently inhibiting key enzymes including FAS and ACC. Furthermore, LCAT interacts with caveolin-1 (CAV1) to inhibit triglyceride (TAG) hydrolysis and with carnitine palmitoyl transferase 1A (CPT1A) to suppress fatty acid oxidation (FAO), ultimately reducing energy availability in hepatocellular carcinoma (HCC) cells (31).

## 4 Conclusion

In this study, *Nannochloropsis gaditana* powder was utilized as the raw material to extract and purify lipids through subcritical extraction combined with molecular distillation, yielding a product enriched in EPA. Animal model experiments demonstrated that *N. gaditana* lipid supplementation significantly attenuates hyperlipidemia-induced increases in body weight, liver weight, and abdominal fat accumulation. Furthermore, Furthermore, improvements in serum and hepatic biochemical profiles corroborated the lipid’s protective effects against hyperlipidemia-associated metabolic disturbances. Overall, the findings of this study provide compelling evidence supporting the potential of *N. gaditana* lipid as a lipid-lowering agent for the management of hyperlipidemia and highlight its promise as a functional food ingredient or as a foundation for the development of anti-hyperlipidemic therapeutics. However, to fully realize its potential in clinical and functional food applications, future research should delve more deeply into the molecular mechanisms through which *N. gaditana* lipids modulate hyperlipidemia-related signaling pathways (such as PPAR-α, SREBP, or LDL receptor pathways) and conduct comprehensive safety assessments of *N. gaditana* lipids.

## Data Availability

The original contributions presented in this study are included in this article/supplementary material, further inquiries can be directed to the corresponding authors.
